# Green synthesis of Ag–Fe bimetallic nanoparticles using fungal filtrates: unlocking multifunctional medical and environmental applications†

**DOI:** 10.1039/d4ra07541b

**Published:** 2025-01-17

**Authors:** Bhakti Patel, Nisha Choudhary, Dushyant Dudhagara, Mudassar Shahid, Rabbani Syed, Virendra Kumar Yadav, Dipak Kumar Sahoo, Ashish Patel

**Affiliations:** a Department of Life Sciences, Hemchandracharya North Gujarat University Patan 384265 Gujarat India bhaktipatel2233@gmail.com nishanaseer03@gmail.com; b Department of Life Sciences, Bhakta Kavi Narsinh Mehta University Junagadh Gujarat India dushyant.373@gmail.com; c Department of Pharmaceutics, College of Pharmacy, King Saud University P.O.Box-2457 Riyadh 11451 Saudi Arabia mahemad1@ksu.edu.sa rsyed@ksu.edu.sa; d Marwadi University Research Center, Department of Microbiology, Faculty of Sciences, Marwadi University Rajkot 360003 Gujarat India; e Department of Veterinary Clinical Sciences, College of Veterinary Medicine, Iowa State University Ames Iowa USA

## Abstract

The current investigation focuses on synthesizing Ag–Fe bimetallic nanoparticles (AgFe-BMNPs) using cell-free filtrates of the *Gymnascella dankaliensis* as a novel fungal reducing agent. The optical, morphological, and surface properties of these fungus-fabricated AgFe-BMNPs and their monometallic counterparts (AgNPs and FeNPs) were analyzed using sophisticated nanotechnology instruments. The UV-visible spectrum showed peaks at 231 nm and 415 nm for BMNPs and 450 nm and 386 nm for AgNPs and FeNPs, respectively. XRD diffractograms revealed crystallographic peaks at 32.96°, 35.32°, and 49.32° for AgFe-BMNPs with crystalline size of 10.68 nm. FTIR spectrum indicates peaks at 954 cm^−1^ (M–O bond) and 599 cm^−1^ (M–C\M–L bond). Agglomerated, spherical BMNPs with a mean size of 96.76 nm were spotted in SEM micrographs. The BMNPs were tested for anticancer and antibacterial activities, dye removal efficiency, and seed germination enhancement. The anticancer study found that AgFe-BMNPs hold promising potential for application in breast cancer therapy with a 1 μg mL^−1^ IC_50_ value. It also exhibited potent antibacterial activity with a 50 μg mL^−1^ concentration against *Bacillus cereus*,*Serratia marcescens*, *Bacillus megaterium*, and *Staphylococcus aureus*. A comparative batch adsorption study for methylene blue dye removal over 180 min showed removal capabilities of 89% for BMNPs. Different concentrations (0.02, 0.04, 0.08 mg mL^−1^) of BMNPs also demonstrated superior efficiency up to 90% enhanced seed germination at the 6 h mark and 91.87% enhanced water retention capacity in *Vigna radiata*. This research underscores the medical, environmental, and agricultural potential of AgFe-BMNPs, highlighting their multifaceted benefits in nanotechnology.

## Abstract 2

Synthesis of Ag–Fe bimetallic nanoparticles using *Gymnascella dankaliensis* filtrates as a novel fungal reducing agent, exploring their diverse applications in medical, environmental, and agricultural fields.

## Graphical abstract

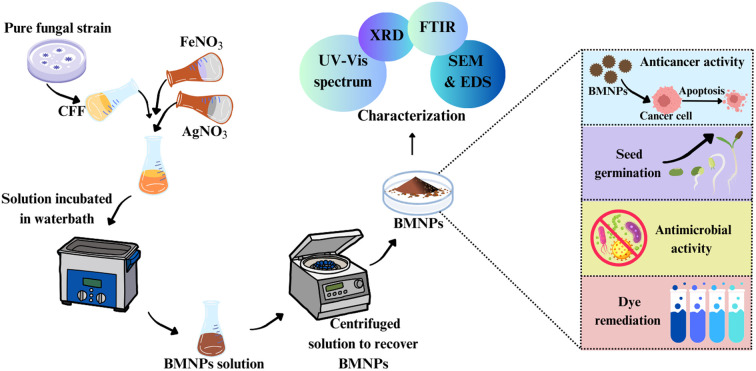

## Introduction

Nanotechnology is a rapidly developing discipline that focuses on manipulating materials from bulk to nanoscale-size particles that are nanoparticles (NPs). A wide variety of nanomaterials (metal oxides, carbon nanotubes, quantum dots, and, most famously, metal NPs) are covered under this study area; for example, metals like silver, iron, gold, *etc.* can be reduced to NPs to enhance their surface-to-area ratio and properties.^1^ Metal nanoparticles, synthesized by reducing metal salts, exhibit unique properties due to their nanoscale dimensions and are considerably more stable. Because of their unique characteristics, they are very interesting for a wide range of applications in various industries, such as environmental remediation, biomedical sciences, and catalysis.^2^

Bimetallic nanoparticles (BMNPs), composed of two different metals, exhibit a wide range of physiochemical properties, making them more useful in diverse applications.^3^ BMNPs can show excellent anticancer activities against various cancer cell lines, as cancer is a significant health challenge faced by humans worldwide. Traditional methods, like chemotherapy and radiation therapy, are effective but cause severe side effects. Among different types of cancers, breast cancer is the most critical malignancy in women. Studies have revealed that BMNPs of AgFe, AuFe, and AuAg may be suitable for breast cancer treatment.^4–7^ The BMNPs, for example, Ag–Fe bimetallic nanoparticles (AgFe-BMNPs), show the advantage of the synergistic interactions between silver and iron, resulting in increasing activities compared to their monometallic NPs. That highlights the significance of investigating BMNPs for their potential ability to provide extra benefits over monometallic NPs.^8^ The enhanced antimicrobial properties of BMNPs can make them effective against various pathogens, including antibiotic-resistant bacteria that cause nosocomial infections. The AgFe and AgCu were reported as potential antimicrobial BMNPs against Gram-positive and Gram-negative bacteria.^9–11^ In environmental applications, BMNPs like AgFe, AgCo, AgNi, and AgCu are widely reported for dye remediation.^12^ Moreover, the studies show seed priming of BMNPs can enhance the seed germination rate and benefit seedling growth.^13^ Three primary methods are majorly used for the fabrication of metal NPs: chemical, biological, and physical. Physical and chemical techniques give exact control over the characteristics of NPs, but they are frequently responsible for challenging circumstances like high energy input and producing hazardous byproducts. On the other hand, the biological method is a more ecologically friendly and sustainable approach. This biological approach reduces harmful environmental impact and conserves energy, aligning with the principles of green chemistry and sustainable development. This research aims to develop innovative strategies for pollution control (environmental remediation), increasing agricultural yields, and medical applications like anticancer activity and controlling the growth of opportunistic pathogens that cause nosocomial infections.^14^ The biological synthesis of NPs utilizes various biological reducing and capping agents such as plant extracts, bacterial cells, algae extracts, or fungal extracts. Microbes are widely distributed and can be easily isolated from any environment. Among these agents, fungi have attracted attention because of their rich bioactive compounds and metabolic capabilities. Fungi exhibit diverse metabolic pathways and adaptability to different environmental conditions, making them ideal candidates for sustainable NPs production.^15^ Fungal species, in particular, are known for their richness in enzymes and secondary metabolites, making them excellent candidates as reducing agents for NPs biosynthesis.

By utilizing fungi, nanoparticles such as copper nanoparticles (CuNPs), iron nanoparticles (FeNPs), silver nanoparticles (AgNPs), gold nanoparticles (AuNPs), platinum nanoparticles (PtNPs), palladium nanoparticles (PdNPs), and selenium nanoparticles (SeNPs) were successfully reported to be synthesized.^16^ Numerous studies have been reported on the use of fungi in the fabrication of AgNPs, as Win and coworkers^17^ observed the AgNPs synthesis mediated by fungi (*Alternaria* sp.) as fungi reduce the Ag^+^ into Ag^0^ in the presence of some or another reducing agents facilitates the synthesis of AgNPs and Mathur^18^ reported biosynthesis of FeNPs by fungi (*Penicillium oxalicum*). AgFe-BMNPs were also biosynthesized by Fuad Ameen^19^ using cell-free filtrate of the fungus *Aspergillus terreus*, which was added into a mixed precursor solution of Ag–Fe salts and kept in a microwave reactor. A diverse range of fungi has been discovered in association with sponges in marine ecosystems. Wang^20^ conducted a review focusing on sponge-associated fungi such as *Penicillium* sp., *Aspergillus* sp., *Trichoderma* sp., and *Gymnascella* sp. Among these fungi, *Gymnascella* sp. is notable due to its considerable abundance as a source of bioactive molecules. Notably, researchers such as Amagata and colleagues^21^ have reported the discovery of bioactive compounds known as gymnastatins A–G, I–K, Q, and R from *Gymnascella* sp. These compounds have been demonstrated to be cytotoxic to cancer cell lines, showcasing their potential as strong cytotoxic agents. Tong and coworkers^22^ chemically evaluated such bioactive substances isolated from *Gymnascella dankaliensis*, and they reported that the production of additional tyrosin-derived alkaloids with promising anticancer effects is primarily dependent on the secondary metabolites dankastatin and gymnastatin. *Gymnascella dankaliensis* is a fungus, and it is classified within the domain Eukarya and the Kingdom fungi, where it belongs to the phylum Ascomycota, class Eurotiomycetes, order Onygenales, and family Gymnoascaceae. The genus is *Gymnascella*, and the species is named *Gymnascella dankaliensis*. In this study, the potential of different fungi for the synthesis of NPs was screened and focused on the synthesis of AgFe-BMNPs and its characterization with high-potential applications in anticancer activity, antibacterial activity, methylene blue dye remediation, and improvement in seed germination of mung bean seeds.

## Materials and methods

### Materials

Pure plates of fungal strain (*Gymnascella dankaliensis*,*Aspergillus quadrilineatus*, and *Talaromyces albobiverticillius*) received from Gujarat Biotechnology Research Centre (GBRC) (Gandhinagar, Gujarat, India). HiMedia (Mumbai, India) provided the Potato Dextrose Agar (PDA), Potato Dextrose Broth (PDB), Nutrient Broth (NB), and Nutrient Agar Medium (NAM), as well as Whatman Filter Paper Grade No. 1, AgNO_3_, FeNO_3_, and methylene blue dye. The source of 3-(4,5-dimethylthiazol-2-yl)-2,5-diphenyltetrazolium bromide (MTT) was Sigma Aldrich, located in St. Louis, Missouri, USA. Thermo-Fisher Scientific (Waltham, MA, USA) supplied Dulbecco's modified eagle medium with high glucose (DMEM-HG), fetal bovine serum (FBS), dimethyl sulfoxide (DMSO), trypsin EDTA, and antibiotics. From the National Centre for Cell Science (NCCS, Pune, India), the MDA-MB-231 cell line was acquired. All the chemicals employed in the study were of high purity and analytical grade, as well as certified *Vigna radiata* seeds.

### Preparation of fungal cell-free filtrate (CFF)

Mater plates of fungal strains received from GBRC were used to prepare the pure plates for that sterile potato dextrose agar (PDA) plates were inoculated using the point inoculation method and incubated at 25 °C for 72 h. Following that, a loop full of fungal spores from a pure plate was used to inoculate potato dextrose broth (PDB), which was then incubated for 72 h at 120 rpm and 25 °C in an incubator shaker. Using Whatman filter paper, the fungal cell mass was filtered after incubation, and the filtrate was discarded. The obtained fungal cell mass was rinsed three times with distilled water, then 8 g (wet weight) was resuspended in 100 mL Milli-Q water and incubated at 60 °C for 24 h. As soon as incubation was completed, the fungal cell mass was filtered, the mycelium-free filtrate was collected, named fungal cell-free filtrate (CFF), and the fungal cell mass was discarded. CFF has been utilized for the fabrication of NPs.^23^

### Screening of fungal strains for the synthesis of NPs

Fungi received from GBRC (*Gymnascella dankaliensis*, *Aspergillus quadrilineatus*, *Talaromyces albobiverticillius*) were evaluated for the fabrication of NPs, the prepared CFF and an aqueous solution of the metal salt precursor was mixed in 1 : 9 ratio and incubated in dark condition for 24 h. CFF of each fungal strain was examined for the synthesis of Ag NPs and FeNPs by mixing with metal salt precursors AgNO_3_ and FeNO_3,_ respectively. This preliminary evaluation was done by detecting visible color changes in the solution using UV-visible spectroscopy.

### Optimization of NPs synthesis

Synthesis of AgNPs, FeNPs, and AgFe-BMNPs was optimized by varying the pH, temperature, and concentration of metal salt precursor. To optimize the pH conditions, the pH levels of CFF were adjusted to 5, 7, and 9. The initial pH of CFF was 7, with pH 5 achieved by adding a few drops of 1 N H_2_SO_4_ solution, while pH 9 was attained by adding a few drops of 1 M NaOH solution. For temperature optimization, the solution was incubated at 25 °C and 30 °C in an incubator and at 60 °C in a water bath. The synthesis of NPs was optimized using AgNO_3_ and FeNO_3_ concentrations of 1 mM, 5 mM, and 10 mM, respectively. The synthesized NPs were then checked for maximum positive results by measuring their weight using a weighing machine.

### Synthesis of NPs

**Fig. 1:**
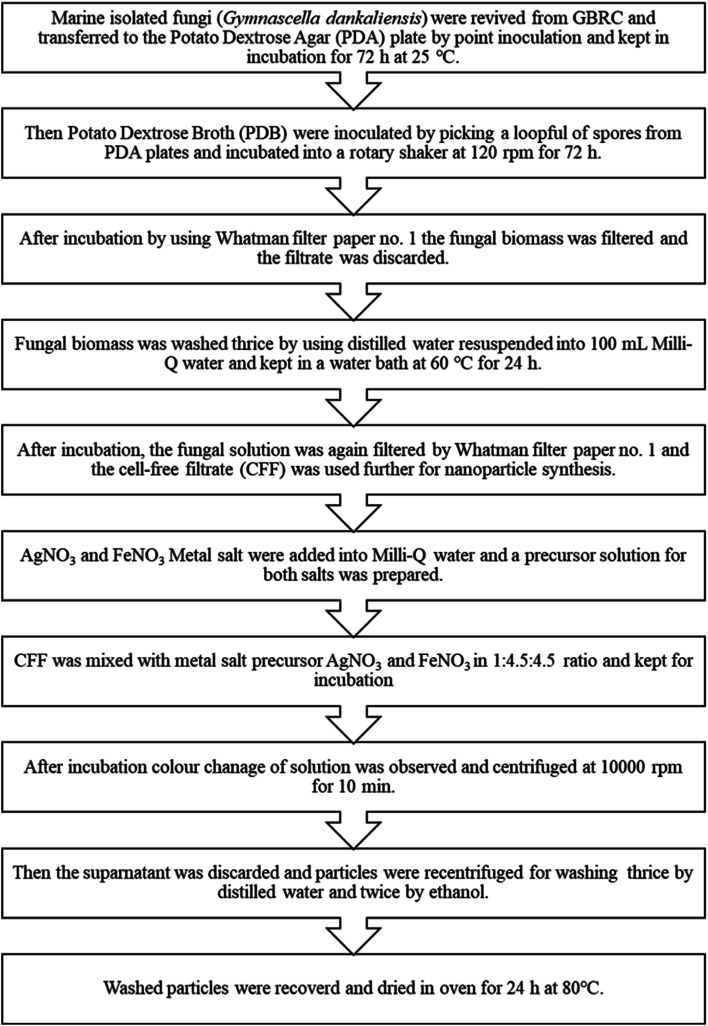
Flow chart of the methodology for AgFe-BMNPs synthesis.

For the synthesis of NPs, firstly 10 mM aqueous solution of AgNO_3_ and FeNO_3_ was prepared. For the synthesis of AgNPs and FeNPs, CFF of *Gymnascella dankaliensis* was used, and an aqueous solution of AgNO_3_ was mixed in a 1 : 9 ratio (CFF : AgNO_3_) and incubated into a water bath at 60 °C for 24 h. Similarly, FeNPs were synthesized by a mixture of CFF and FeNO_3_ in the same ratio used before. AgFe-BMNPs were synthesized by adding CFF with AgNO_3_ and FeNO_3_ in a 1 : 4.5 : 4.5 ratio (CFF : AgNO_3_ : FeNO_3_) and incubated at the same conditions. The derived NPs were then recovered by centrifuging the incubation solution at 10 000 rpm for 10 min. Then the supernatant was discarded and the pellets were washed thrice with distilled water, followed by centrifugation, then washed twice with ethanol and centrifuged. Finally, the pellets were collected into a dry Petri dish and kept in a hot oven at 80 °C overnight. The powder was then collected the next day and used for characterization (Fig. 1).

### Characterization of NPs

NPs were characterized using several kinds of methods, including UV-visible spectrophotometer, FTIR, XRD, SEM, and energy dispersive X-ray spectroscopy equipment. UV-visible measurements were carried out in the range of 200–800 nm at a resolution of 1 nm using a LABMAN LCD LMSP-UV1900 double-beam UV-visible spectrophotometer. The UV-visible spectra of monometallic NPs and BMNPs were taken by dissolving about 1 mg of NPs in deionized water and then sonicated for 10 min.^24^ To perform the FTIR study of NPs produced from *Gymnascella dankaliensis*, transmission measurements were taken from the mid-IR range 599–4000 cm^−1^ with a resolution of 2 cm^−1^. FTIR spectra were produced by forming a pellet with KBr and analyzing them using an S6500 Spectrum instrument (PerkinElmer, USA).^25^ The crystalline and amorphous phase patterns of NPs were measured using an advanced D-8 (Bruker, Netherlands) instrument-equipped X'celerometer. XRD patterns were obtained in the 2-theta range of 20–70 with a step size of 0.02 and a time of 5 seconds per step at 40 kV voltage and a current of 30 mA, and crystalline size was measured by using Scherrer's formula^26,27^ The morphological study of NPs was performed using the Apreo LoVac Novo Nanosem, FEI (USA). The NPs powder samples were put onto carbon tape and placed on an aluminum stub holder. The material was examined using the gold sputtering technique.^28^ The energy dispersive X-ray spectroscopy (EDS) of NPs was studied by an Oxford EDS analyzer linked to the FESEM at varied magnifications and 20 kV.^29^

### Anticancer activity of AgFe-BMNPs

The anticancer efficiency of AgFe-BMNPs was evaluated using an MTT assay.^30^ Human breast cancer cells (MDA-MB-231) were grown in DMEM-HG media supplemented with 10% FBS and 1% antibiotic. After achieving 80% to 90% confluence, cells were planted at a density of 7000 per well in 96-well culture plates. The next day, cells were treated with different doses (0.1, 0.5, 1.0, 5.0, and 10 μg mL^−1^) of AgFe-BMNPs for 24 h and 48 h. After a while, cells were treated with MTT reagent (5 mg mL^−1^) for 4 h. Then, 100 μl DMSO was incubated for 15 min to dissolve the formazan crystals. The absorbance was measured at 570 nm using a multimode plate reader (Synergy H1, BioTek, USA), and the percentage of cell viability was determined.

### Antibacterial activity

Antibacterial activity of 50 μg mL^−1^ AgNPs, FeNPs, and AgFe-BMNPs solution was checked against a few bacterial strains (*Bacillus cereus*, *Serratia marcescens*, *Bacillus megaterium*, *Staphylococcus aureus*) by disc diffusion method for that bacterial active cultures were prepared by inoculating loopful of culture from a pure plate of bacteria into nutrient broth and kept into incubator shaker at 37 °C, 120 rpm for 24 h. After incubation, the nutrient agar plates were swabbed with active cultures, and the discs loaded with Ag, Fe monometallic NPs, and AgFe-BMNPs were placed on them and incubated at 37 °C for 24 h. After the incubation, the zone of inhibition was measured.

### Preparation and remediation of aqueous solution of methylene blue dye

Methylene blue (MB) dye stock solution (100 ppm) was prepared by adding 10 mg in 100 mL Milli-Q water, and the stock solution was further diluted to have a 10 ppm concentration. To check the MB dye remediation 5 mg of AgFe-BMNPs were added into 40 mL of 10 ppm MB dye solution and kept on a magnetic stirrer. The readings at intervals of 10 min were taken by using a UV-vis spectrophotometer of the solution. The dye removal percentage was calculated by using the formula:
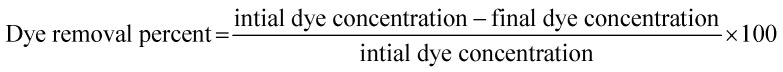


### Seed germination activity

Three different concentrations (0.2 mg, 0.4 mg, and 0.8 mg) of each AgNPs, FeNPs, and AgFe-BMNPs were taken for the seed germination study. Then, these NPs were separately suspended in each labeled tube containing 10 mL Milli-Q water. Additionally, a control test tube containing only 10 mL Milli-Q water was also prepared. All test tubes were sonicated for 10 min to ensure proper dispersion of NPs. On the other hand, sets of certified mung bean seeds (*Vigna radiata*), each containing 10 seeds, were accurately weighed and washed thrice with distilled water. One set of seeds was added to each respective NPs solution test tube and the control tube, allowing them to soak for 1 h. After the soaking period, the seeds were transferred to sterile Petri plates containing a thin layer of sterile cotton for germination. Observations were made periodically, with germination progress recorded at 2 h intervals to monitor and compare the effects of different NPs concentrations and compositions on seed germination rates. To calculate the water content percentage of the seeds treated with AgFe-BMNPs, AgNPs, and FeNPs, the following formula was used:
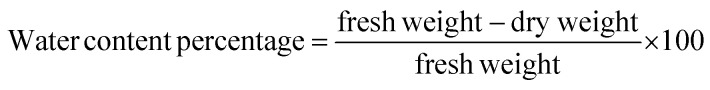


All the experimental sets were performed in triplicates to ensure the accuracy of the results.

## Results and discussion

### Screening of ideal fungal strains for NPs synthesis

The comparative study of NPs synthesis revealed interesting findings using the CFF extracted from different fungi was primarily evaluated by detecting color changes from pale yellow to blackish brown of the solution using UV-visible spectroscopy. When CFF of *Gymnascella dankaliensis* was mixed with AgNO_3_ and FeNO_3_ precursor, it successfully synthesized AgNPs and FeNPs (Table 1). While, NPs synthesis using CFF from *Aspergillus quadrilineatus* yielded positive results with AgNO_3_ precursor, synthesizing AgNPs while showing negative outcomes with FeNO_3_, indicating the inability to synthesize FeNPs. In the same way, CFF derived from *Telluromyces albobiverticillius* exhibited positive results with AgNO_3_ precursors, producing AgNPs, but failed to synthesize FeNPs.

**Table 1:** Screening of fungal strains for the synthesis of NPs^a^

Name of fungal spp.	Metal salts	
	AgNO_3_	FeNO_3_
*Gymnascella dankaliensis*	+	+
*Aspergillus quadrilineatus*	+	−
*Talaromyces albobiverticillius*	+	−

a Results: +: positive; −: negative.

These results indicate the CFF can reduce and stabilize metal ions into NPs and the inability to reduce some NPs. These results highlight the species-specific reducing capabilities of fungal CFFs towards different metal ions and the importance of fungal species selection in NPs synthesis processes.

### Optimization study of nanoparticles

For the optimization study, temperature variations (25 °C, 37 °C, and 60 °C), pH (5, 7, and 9), and precursor solution concentration (1, 5, and 10 mM) were explored (Table 2). The findings demonstrate that conditions of 60 °C temperature, pH 9, and 10 mM concentration lead to increased synthesis of both Ag and FeNPs compared to other tested conditions, as the obtained particles exhibited greater weight.

**Table 2:** Optimized conditions for the synthesis of Ag and FeNPs^a^

Precursor solution	Various condition								
	Temperature °C			pH			Concentration of precursor in mM		
	25 °C	37 °C	60 °C	5	7	9	1 mM	5 mM	10 mM
Ag	+	+	+++	−	+	+++	+	++	+++
Fe	+	+	++	−	+	+++	+	+	++

a Results: +: less positive; ++: moderate positive; +++: highly positive; −: negative.

The highly positive results indicate the maximum mass of NPs was synthesized. These optimized parameters demonstrate enhanced NPs synthesis efficiency and highlight the importance of precise control over temperature, pH, and precursor concentration to synthesize AgNPs and FeNPs successfully.

### UV-vis spectroscopy analysis

**Fig. 2:**
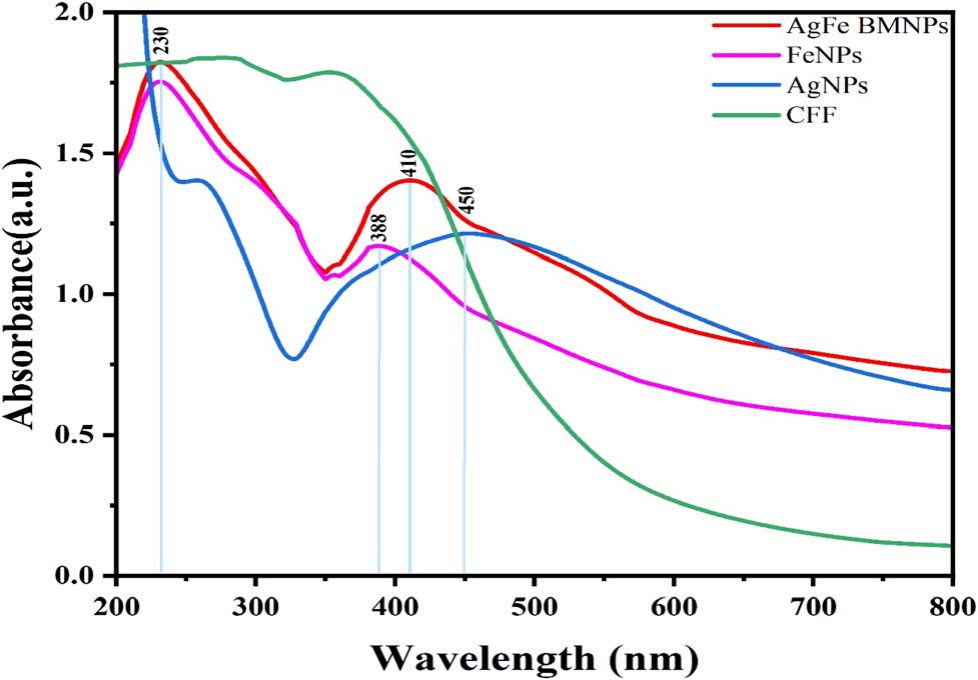
Absorbance spectrum of AgNPs (450 nm), FeNPs (230 nm and 388 nm), AgFe-BMNPs (230 nm and 410 nm), and CFF of *Gymnascella dankaliensis* for UV-visible spectroscopy analysis.

The UV-visible spectroscopy investigation revealed distinctive peaks for each NPs sample. The AgNPs displayed a peak at 450 nm, while the FeNPs graph showed a peak at 230 nm and another at 388 nm. Remarkably, the AgFe-BMNPs sample showed peaks at 230 nm and 410 nm, demonstrating distinct optical features associated with the presence of AgNPs and FeNPs in the bimetallic formulation (Fig. 2).

These spectral properties provide essential information into the composition and optical behavior of each NPs sample, contributing to a better understanding of its possible applications in diverse areas. Cruz *et al.*^8^ found that the absorption spectrum of the BMNPs was comparable to that of FeNPs, indicating the formation of silver–iron core–shell BMNPs. The UV-visible spectrum with a maximal absorbance peak at 425 nm for AgNPs, while a peak at 290 nm and a small peak at 350 nm was obtained for FeNPs and Ag–Fe BMNPs synthesized by *G. jasminoides* leaves extract. Comparable findings by Asfar *et al.*^31^ have been reported that the UV-visible absorbance spectrum of the synthesized Ag–Fe BMNPs from date fruit shows a peak below 300 nm for FeNPs, at 450 nm for AgNPs. The presence of a peak below 300 nm, the same as FeNPs, but the absence of a peak at 450 nm concluded the formation of a core–shell of Ag–Fe bimetallic. Similar experiments conducted by Zhang *et al.*^32^ have found that the peak associated with AgNPs often lies between 390 and 580 nm. Comparing AgFe BMNPs synthesized using CFF of *Gymnascella dankaliensis* with other studies shows metals successfully reduced to form BMNPs and dual peaks were represented due to their synergetic effect.

### X-ray diffraction (XRD)

**Fig. 3:**
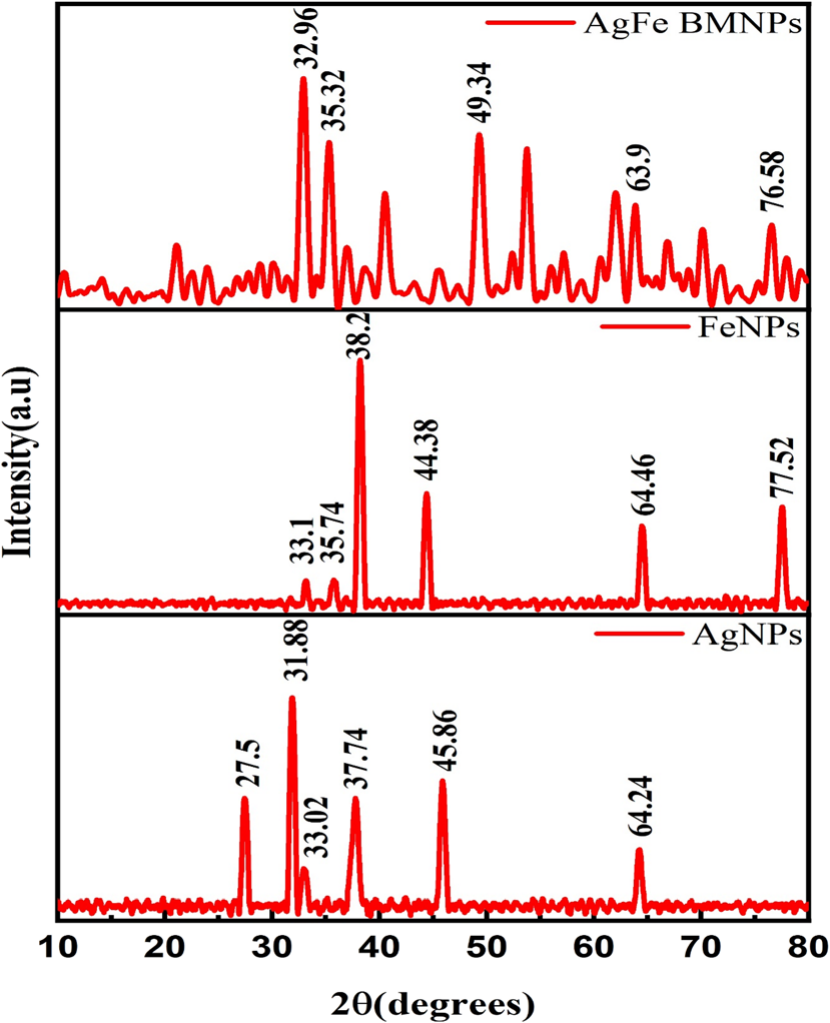
Diffractogram of AgFe-BMNPs, Ag, and FeNPs.

The XRD analysis results (Fig. 3) show distinct peak patterns for FeNPs, AgNPs, and AgFe-BMNPs. FeNPs exhibited major peaks at 33.1°, 35.74°, 38.2°, 44.38°, 64.46°, and 77.52°, while AgNPs displayed major peaks at 31.88°, 27.5°, 33.02°, 37.74°, 45.86°, and 64.24°. For AgFe-BMNPs, major peaks were observed at 32.96°, 35.32°, 49.32°, and 63.90°, which corresponds to (111), (200), (220), (311), and (222) lattice planes. According to Malik *et al.*,^33^ these planes highlight the face-centered cubic (fcc) structure of silver particles. The XRD patterns of AgFe-BMNPs exhibit diffraction peaks consisting of Ag and Fe, indicating the creation of bimetallic phases comprising these two elements. A similar study by Kamli *et al.*^34^ observed major diffraction peaks for AgNPs and Ag–Fe BMNPs at 38.01, 44.21, 64.7, and 77.49°, while for FeNPs, major diffraction peaks at 44.21 and 64.7°.

These distinctive peak positions indicate the crystalline nature of the AgFe-BMNPs and provide an understanding of their structural characteristics. Additionally, the crystallite size was determined to be 10.68 nm for AgFe-BMNPs, 11.73 nm for FeNPs, and 12.21 nm for AgNPs, indicating the nanoscale dimensions of these materials, they have significance for their numerous applications in nanotechnology and materials research.

### Fourier transform infrared spectroscopic (FT-IR) analysis

**Fig. 4:**
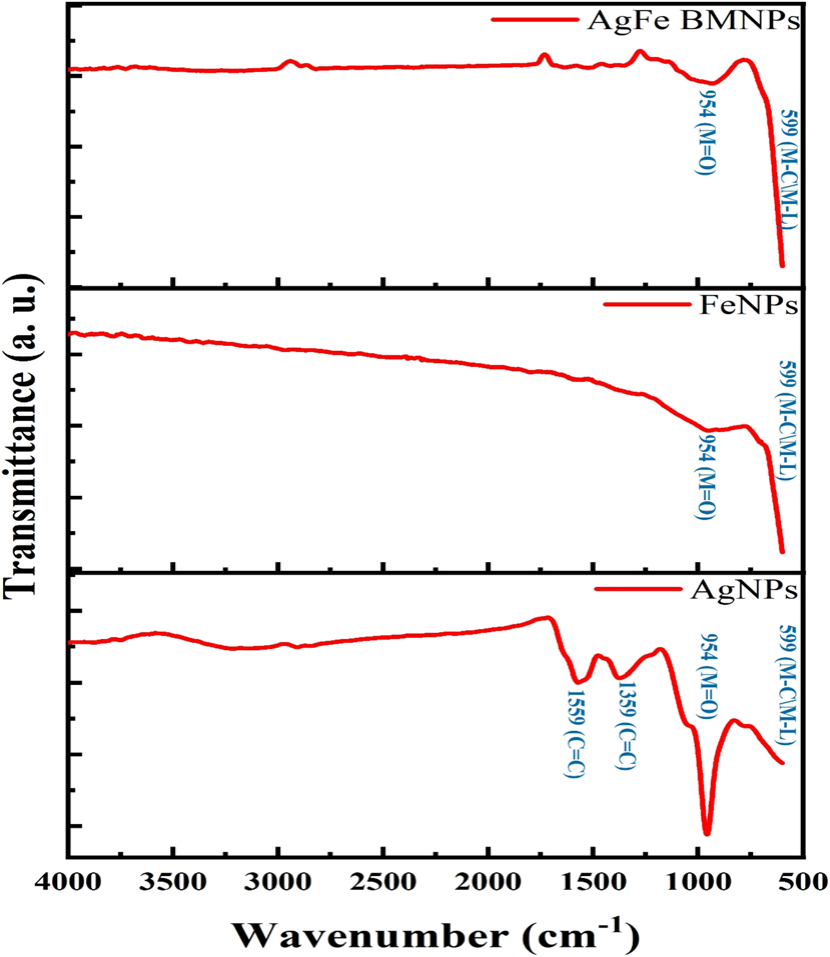
FTIR spectrum for AgFe-BMNPs, Ag, and FeNPs.

The FTIR analysis of NPs samples provided a valuable understanding of their chemical compositions and surface functionalization through specific bonding regions corresponding to functional groups (Fig. 4). AgFe-BMNPs exhibited characteristic bonding peaks at 954 cm^−1^ (indicating metal–oxygen bonds) and 599 cm^−1^ (suggestive of metal–ligand vibrations or metal-carbon bonds), reflecting the presence of elements.^35^ The observed peaks likely suggest interactions between AgFe-BMNPs and bioactive substances, such as peptides and amino acids, present in the fungal CFF. These interactions play a vital role in both stabilizing the nanoparticles and enhancing their functional characteristics. FeNPs displayed similar bonding patterns, supporting the presence of iron and associated metal–ligand or metal-carbon interactions. AgNPs containing silver exhibited distinctive peaks at 1559 cm^−1^ and 1359 cm^−1^ (C=C bonds), 954 cm^−1^ (possible metal–oxygen bonds), and 599 cm^−1^ (metal–ligand or metal–carbon bonds), highlighting its unique surface chemistry.^36^

These analyses explain key information about the structural characteristics and functional groups present on the NPs surfaces can be due to the use of novel CFF of the fungus *Gymnascella dankaliensis*, which can enhance their properties and applications in diverse fields like nanotechnology and materials science, including catalysis, and biomedical applications.

### Morphological studies (SEM-EDX)

**Fig. 5:**
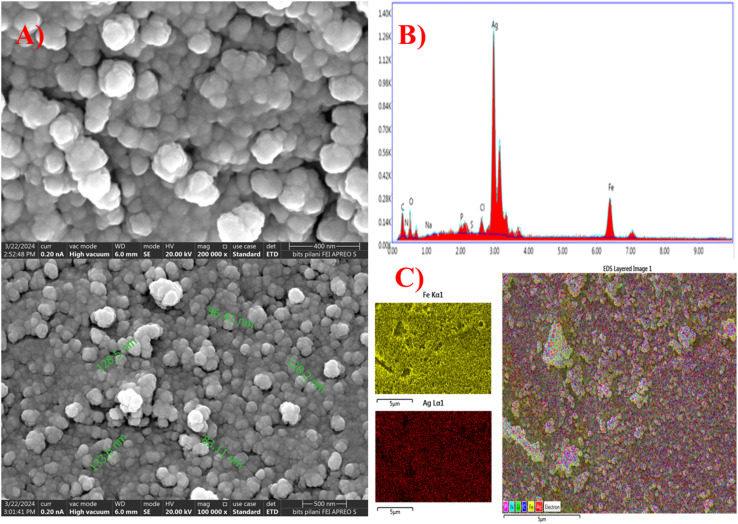
SEM images (A), EDX spectra (B), and elemental mapping (C) of AgFe-BMNPs.

FE-SEM analysis revealed spherical agglomerated AgFe-BMNPs with a mean size of 96.76 (Fig. 5A). AgNPs also displayed a spherical morphology with an average size of 90.57 nm. At the same time, FeNPs presented an agglomerated and spherical form, ranging from 82.69 nm.

In a similar study conducted by Ameen,^19^ SEM analysis images revealed that Ag–Cu BMNPs synthesized by the fungus *Aspergillus terreus* were agglomerated and spherical. Also, Kamli *et al.*^34^ have reported agglomerated spherically clustered NPs having foam-like projections. These distinct size characteristics within agglomerated states can significantly impact surface area, reactivity, and suitability for diverse applications. The observed morphology highlights the critical role of synthesis parameter control in synthesizing NPs sizes and structures (Fig. 5B and C), displays the elemental composition of AgFe-BMNPs where a peak for Ag, Fe, O, C, N, P, and S can be seen with % weightage of 63.3, 18.5, 11.2, 2.3, 0.8, 1.6, and 0.6, respectively. The presence of C, N, and P could be because of the constituent elements of the fungal extract.

### Anticancer activity of AgFe-BMNPs

**Fig. 6:**
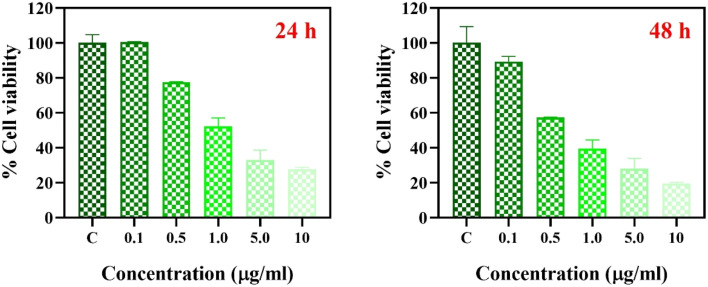
The MTT assay used to determine AgFe-BMNPs *in vitro* anticancer activity. MDA-MB-231 cells were treated with different concentrations of AgFe-BMNPs (0.1, 0.5, 1.0, 5.0, and 10 μg mL^−1^) for 24 and 48 h.

The anticancer activity of AgFe-BMNPs was evaluated against breast cancer cells using the MTT assay (Fig. 6), showing that the compound's cytotoxicity towards MDA-MB-231 cells was concentration- and time-dependent. At 1 μg mL^−1^ concentration, cell viability was 52.2% ± 3.8% after 24 h and 39.4% ± 4.1% after 48 h. The MTT assay results show that a 1 μg mL^−1^ concentration resulted in about 50% cell death. Hence, it has IC_50_ as 1 μg mL^−1^, even after 24 h, indicating the anticancer potential of AgFe-BMNPs.

Similar results were observed by MTT assay against MCF-7 and HepG-2 cells. Thirupathi^37^ noted the IC_50_ values of Se–ZnO NPs to be 67.9 μg mL^−1^ and 79.15 μg mL^−1^, respectively. The Ag–ZnO NPs synthesized by Hashem & El-Sayyad^38^ revealed IC_50_ values for MCF7 and Caco2 cells were 104.9 and 52.4 μg mL^−1^, respectively, conforming anticancer activities.

### Antibacterial analysis

**Fig. 7:**
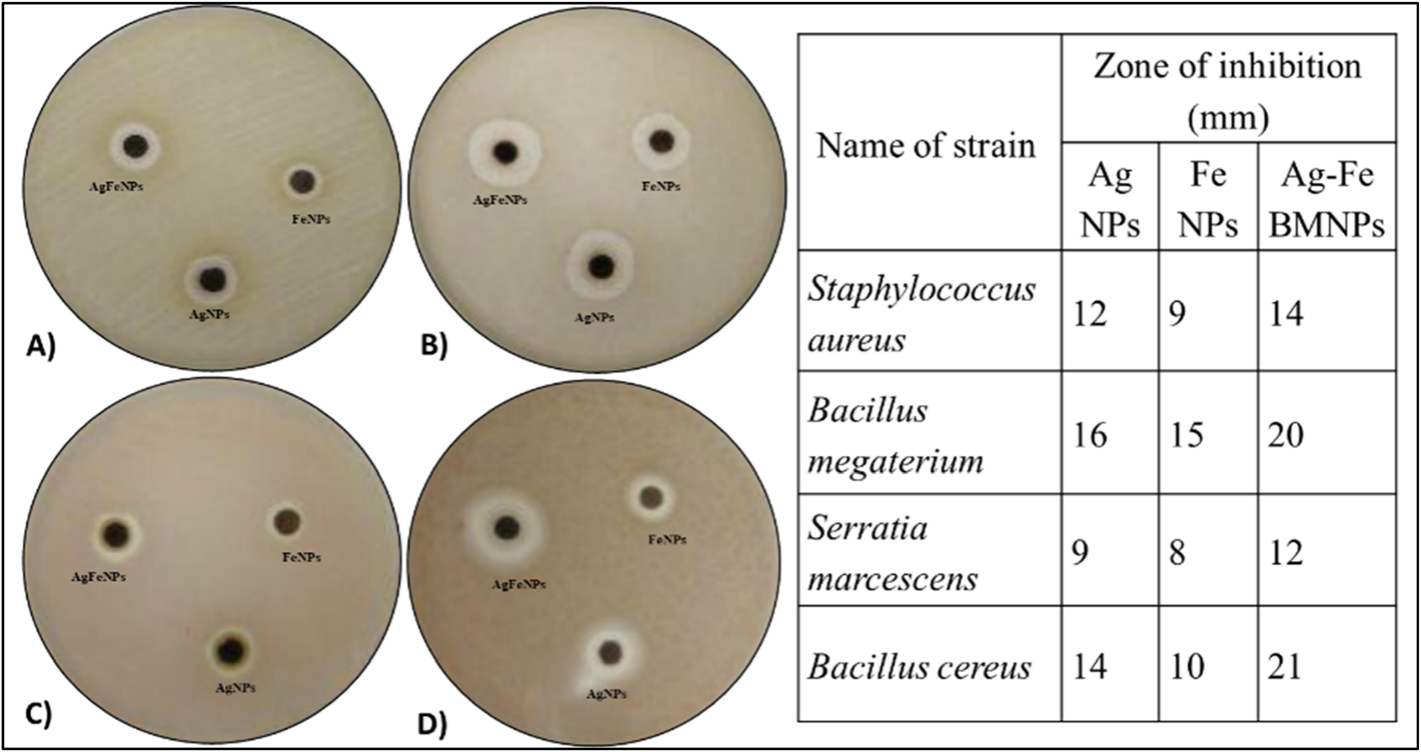
Zone of inhibition produced by 50 μg mL^−1^ of different NPs against (A) *Staphylococcus aureus*, (B) *Bacillus megaterium*, (C) *Serratia marcescens*, (D) *Bacillus cereus*.

The antibacterial activity analysis revealed varying degrees of inhibition zones against different bacterial strains for different NPs (Fig. 7). Specifically, inhibition zones were observed for *Staphylococcus aureus* at 9 mm for FeNPs, 12 mm for AgNPs, and 14 mm for AgFe-BMNPs.

Conversely, *Bacillus megaterium* exhibited inhibition zones of 15 mm for FeNPs, 16 mm for AgNPs, and 20 mm for AgFe-BMNPs. *Serratia marcescens* showed inhibition zones of 8 mm for FeNPs, 9 mm for AgNPs, and 12 mm for AgFe-BMNPs. Finally, inhibition zones were recorded for *Bacillus cereus* at 14 mm for AgNPs, 10 mm for FeNPs, and significantly larger at 21 mm for AgFe-BMNPs. The antibacterial activity conducted by Murtaza *et al.*^39^ used 100 mg mL^−1^ AgFe-BMNPs against *K. pneumonia*, *E. coli*, and *S. aureus* and observed a 1 cm zone of inhibition against all bacterial species. Similarly, Sandupatla *et al.*^40^ found FeAg BMNPs exhibiting 7 mm and 4 mm activity against *Escherichia coli* and *Bacillus subtilis*, respectively. These results suggest the enhanced antibacterial efficacy of AgFe-BMNPs compared to Ag and FeNPs alone across multiple bacterial strains, highlighting their potential in antibacterial applications.

### MB dye remediation

**Fig. 8:**
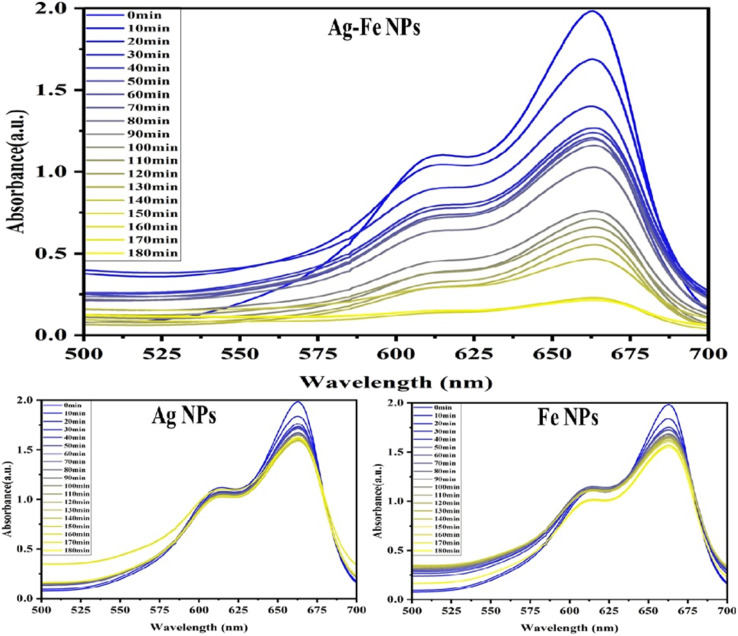
Uv-visible absorption spectrum of MB dye remediation by AgFe-BMNPs treated solution, AgNPs treated solution, and FeNPs treated solution.

**Fig. 9:**
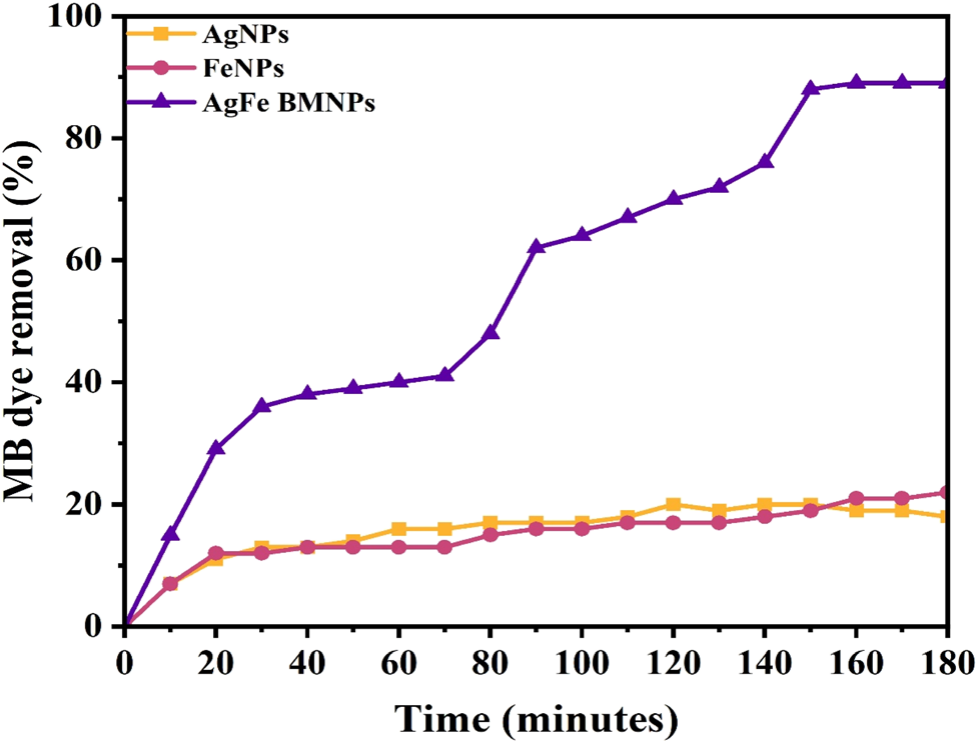
Percent removal graph of MB dye.

The dye remediation results using different NPs reveal varying efficiencies over time. AgFe-BMNPs showed the most promising performance, initially removing 15% of the dye within 10 min and reaching a peak removal rate of 89% at 160 min, maintaining this level until 180 min (Fig. 8 and 9). In contrast, AgNPs showed a lower initial removal of 7% in 10 min and a maximum of 20% removal in 120 min. FeNPs also displayed an initial 7% removal in 10 min but achieved a maximum of 22% removal at 180 min.

The catalytic potential of Cu–Ag BMNPs was examined by Ali *et al.*^41^ for the degradation of Rh–B, MB, and MO dyes. Where more than 90% degradation was recorded for each dye. A remarkable study conducted by Idris & Roy^33^ observed that Ag–Fe_2_O_3_ BMNPs synthesized from cabbage peels exhibited 78% degradation of phenol red dye within 8 min. Similarly, Sudhakar *et al.*^42^ confirmed the 91.23% photocatalytic degradation of malachite green (MG) dye within 180 min by PS-Ag@Fe BMNPs in direct sunlight irradiation. These findings highlight the superior effectiveness of AgFe-BMNPs in long-term dye remediation, followed by FeNPs, while AgNPs fall behind in terms of efficiency and duration of action.

### Seed germination and water content

**Fig. 10:**
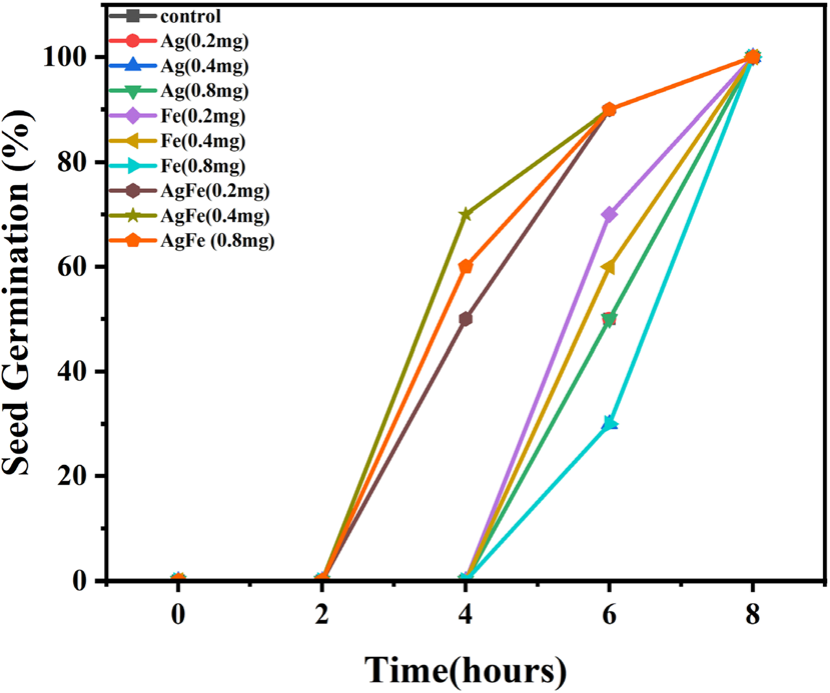
Effect of AgFe-BMNPs and their counterparts on seed germination.

The results obtained by the treatment of AgFe-BMNPs for seed germination activities involved monitoring germination progress from seed sowing until 100% germination was achieved (Fig. 10).

Germination readings were recorded at 2 h intervals, starting from sowing. No germination was observed until the 4 h mark, but significant changes were noted afterward. At the 4 h mark, germination percentages of AgFe-BMNPs at different concentrations (50%, 70%, and 60% for 0.2, 0.4, and 0.8 mg, respectively), whereas at the 6 h mark, the control shows (50%), AgNPs (50%), FeNPs (70%) germination percentage. Subsequently, at 6 h, germination percentages increased to 90% for AgFe-BMNPs across all concentrations, indicating their favorable impact on germination rates. Finally, after 8 h, 100% germination was observed across all treated groups, showcasing the effectiveness of AgFe-BMNPs in promoting seed germination compared to Ag and FeNPs alone, as well as the control group (Table 3).

**Table 3:** Water content percentage of *Vigna radiata* seeds treated with AgFe-BMNPs, Ag, and FeNPs

Treatments		Water content (%)
Control	0 mg/10 mL	91.47
Ag	0.2 mg/10 mL	88.73
	0.4 mg/10 mL	86.91
	0.8 mg/10 mL	87.59
Fe	0.2 mg/10 mL	90.81
	0.4 mg/10 mL	89.88
	0.8 mg/10 mL	88.62
Ag–Fe	0.2 mg/10 mL	91.87
	0.4 mg/10 mL	90.72
	0.8 mg/10 mL	90.31

The fresh weight and dry weight of the seeds were measured, as mentioned in Table 3. The highest water content percentage was observed in seeds treated with 0.2 mg of AgFe-BMNPs, while slightly lower percentage values were recorded for the control group. This finding suggests that the application of AgFe-BMNPs enhanced water retention in the seeds compared to the untreated control group. This indicates the capability of AgFe-BMNPs to improve seed hydration and may have a beneficial impact on seed germination and growth processes. These results highlight the importance of NPs treatments in agricultural applications for enhancing plant performance.

## Conclusions

The study successfully synthesized and characterized NPs using fungal cell-free filtrates (CFF) from *Gymnascella dankaliensis*, highlighting their potential in dye remediation, seed germination enhancement, and antimicrobial activities. *Gymnascella dankaliensis* CFF was particularly effective in synthesizing silver (Ag) and iron (Fe) NPs, with optimal synthesis conditions identified as 60 °C, pH 9, and 10 mM precursor concentration. UV-vis spectroscopy, XRD, and FTIR analyses confirmed the NPs' unique optical properties, crystalline nature, and surface functional groups. FE-SEM morphological studies revealed a spherical shape and size distribution of the NPs. The anticancer study suggests that AgFe-BMNPs hold promising potential for application in breast cancer therapy. AgFe-BMNPs demonstrated superior performance in enhancing seed germination rates and exhibited strong antibacterial activity against various bacterial strains. Additionally, AgFe-BMNPs showed efficient dye remediation capability, removing 89% of methylene blue dye in 180 min compared to both Fe (22%) and Ag (20%) NPs. These findings highlight the significant potential of AgFe-BMNPs in environmental, agricultural, and medical applications, showcasing their multiple benefits in nanotechnology.

## Data availability

Data is contained within the article and ESI.†

## Conflicts of interest

The authors declare no conflict of interest.

## Supplementary Material

RA-015-D4RA07541B-s001
